# Association between caregiver type and catastrophic health expenditure among households using inpatient medical services: using Korean health panel

**DOI:** 10.1186/s12913-023-09703-1

**Published:** 2023-07-03

**Authors:** Yu shin Park, Hyunkyu Kim, Il Yun, Eun-Cheol Park, Suk-Yong Jang

**Affiliations:** 1 Department of Public Health, Graduate School, Yonsei University, 50 Yonsei-to, Seodaemun-Gu, Seoul, 03722 Republic of Korea; 2 Institute of Health Services Research, Yonsei University, Seoul, Republic of Korea; 3 Department of Preventive Medicine, Yonsei University College of Medicine, Seoul, Republic of Korea; 4 Department of Healthcare Management, Graduate School of Public Health, Yonsei University, Seoul, Republic of Korea

**Keywords:** Catastrophic health expenditure, Formal caregiver, Informal caregiver, Comprehensive nursing service

## Abstract

**Background:**

Caregiving services often place a financial burden on individuals and households that use inpatient medical services. Consequently, this study aimed to examine the association between the type of caregiver and catastrophic health expenditure among households utilizing inpatient medical services.

**Methods:**

Data were extracted from the Korea Health Panel Survey conducted in 2019. This study included 1126 households that used inpatient medical and caregiver services. These households were classified into three groups: formal caregivers, comprehensive nursing services, and informal caregivers. Multiple logistic regression was used to analyze the association between caregiver type and catastrophic health expenditure (CHE).

**Results:**

Households receiving formal caregiving had an increased likelihood of CHE at threshold levels of 40% compared to those who received care from family (formal caregiver: OR 3.11; CI 1.63–5.92). Compared to those who received formal caregiving, households using comprehensive nursing services (CNS) had a decreased likelihood of CHE (CNS: OR, 0.35; CI 0.15–0.82). In addition, considering the economic value associated with informal care, there was no significant relationship between households received formal caregiving and informal caregiving.

**Conclusion:**

This study found that the association with CHE differed based on the type of caregiving used by each household. Households using formal care had a risk of developing CHE. Households using CNSs were likely to have a decreased association with CHE, compared to households using informal and formal caregivers. These findings highlight the need to expand policies to mitigate the burden on caregivers for households forced to use formal caregivers.

**Supplementary Information:**

The online version contains supplementary material available at 10.1186/s12913-023-09703-1.

## Background

Nursing care delivery systems differ based on national policies, culture, and healthcare systems in each country. Formal or informal caregivers, such as family members, can reside in hospital rooms to support daily patient care in several Asian countries, including Korea, China, and Taiwan, and some African countries [1].

South Korea maintained a patient–individual caregiver nursing system since the early 1980s [2]. This is a phenomenon in which part of the nurses’ work has been transferred to caregivers due to the lack of nursing staff in Korea [3].

In South Korea, there are usually two types of private caregiving: formal caregivers who receive payment for patient care services during hospitalization, and informal caregivers, called family caregivers, who provide care to family and friends without receiving payment. Approximately 13.8–25.5% of inpatients hire formal caregivers due to the lack of caregivers owing to nuclear familization and the expansion of women’s socioeconomic participation [4–6]. A previous study highlighted that this expansion of private caregiving services resulted in social costs, creating an economic burden on caregiver service costs, or causing the cost of losing family productivity, reaching seven to eight trillion won annually in 2018 [4, 7]. The burden and stress experienced by formal caregivers widely plague South Korea’s healthcare system and have been described in studies of quality of life and out-of-pocket costs for caregiving from conditions such as dementia, Parkinson’s disease, and cancer [8–10]. The issue of informal caregiver burden was explained as a loss of productivity of family members or mental health from conditions such as stroke and cancer [11–13]. However, no studies simultaneously compared the burdens of informal and formal care.

To alleviate the burden on caregivers, the Korean government launched a comprehensive nursing care service (CNS) system in 2015 that provides comprehensive nursing care by nurses and nursing assistants without a protector or caregiver residing for the care of inpatients [4, 14]. The system was expected to decrease the burden of care stress and alleviate the burden of caring costs by paying a CNS instead of an average of 100 dollars per day when a patient is admitted to the CNS ward [14, 15]. However, few studies have evaluated the decrease in the care burden of this service.

This study used a cross-sectional design based on the type of caregiver to evaluate whether the type of care was associated with catastrophic health expenditure. Additionally, this study also investigated whether the type of care was related to the economic burden of households using the CNS, implemented to alleviate the burden of care.

## Methods

### Data sources

This study extracted data from the first wave (2019) of the Korean Health Panel (KHP) dataset. The KHP was conducted by the Korea Institute for Health and Social Affairs and the National Health Insurance Corporation. The KHPS is a national public database (https://www.khp.re.kr) that includes an identification number for each household and member. However, the number is not associated with any personal identifying information, and the data collection system and database were designed to protect respondents’ confidentiality. The KHP includes secondary data to gather and provide household and individual-level scientific data on health service use, expenditure, and health behaviors. The KHP questionnaires consisted of household and member components and were surveyed using both interview and diary methods to supplement memory.

### Participants

We analyzed the household level and extracted households that even one of the household members has been hospitalized and utilizing private caregiving service (formal care, informal and CNS). The data included 6,748 households and 16,589 household members from 2019. First, we extracted 1,495 households out of a total of 6,748 households in which at least on member of household experienced hospitalization during the study period. And of these, 369 households, which used inpatient medical services but did not use caregivers, were excluded. Finally, we extracted 1,126 households for our study (Appendix Figure 1). This was done to minimize the difference in severity between patients using caregivers and those not using caregivers.

### Variables

The dependent variable was CHE, as defined by the World Health Organization (WHO). Xu et al*.* [16] described CHE as when the annual health spending of a household exceeds 40% of the total annual household spending of food expenditure, excluding dining-out expenses [17, 18]. Out-of-pocket health expenditure (OOPHE) refers to payments made on direct health expenditure (households for doctor’s consultation fees, hospital bills, purchase of medication, and emergency medical costs) and indirect health expenditure (caregiver costs). The method of calculating caregiving costs differs depending on the type of caregiving cost. Formal caregiver costs were measured by multiplying the average formal caregiver payment per day by the total number of days that the caregiving services were received. Comprehensive nursing service costs were included in hospital expenses, namely, direct health expenditures. Informal caregiving was excluded from the cost estimation because it did not incur costs. However, there have been discussions on replacing the costs of informal care. Excluding the cost of informal care in economic evaluations and cost of illness studies could lead to underestimating the true costs, benefits, and burdens of interventions and diseases. Therefore, it is crucial to include these costs to ensure a comprehensive range of estimates [19]. Therefore, this study added a sensitivity analysis by calculating costs using opportunity and alternative cost approaches [20, 21]. The opportunity cost approach applied informal care to the 2020 reservation wage rate which was calculated by the cost based on the minimum wage announced by the Minimum wage council republic Korea [22]. The cost was estimated 68,720 won. And the alternative cost approach applied the annual average formal caregiving cost. The average per-day payment to caregivers, used to estimate the cost of daily informal caregiving, was based on the 2018 Healthcare Experience Survey [23]. The OOPHE consisted of household-level data, and one value was allocated to each household characteristic in this study.

The independent variable was the caregiving type, and the study households were categorized into three groups: household members who received care from formal caregivers who were paid for their service (formal caregivers), household members who received comprehensive nursing services (comprehensive nursing services), and household members who received care from informal caregivers, who were family or friends, usually without payment (informal caregivers). In this study, households were classified as “formal caregivers if their members received formal care.” Households were classified as “comprehensive nursing service (CNS)” if their members never received formal care but received comprehensive nursing services.

This study controlled for covariates, such as sociodemographic and socioeconomic factors of the household. Sociodemographic factors included householders’ sex (male or female), householders’ age (< 65, 65–74, or > 65 years), number of household members (1,2, or ≥ 3), the existence of older adults in a household (yes or no), and region (urban or rural). Socioeconomic factors included income quartile (low, low-middle, middle-high, or high), having a medical aid health coverage scheme (yes or no), and householders’ employment status (paid worker, self-employed worker, or other). Inoccupation, students, and unpaid family workers were classified as “other.” Furthermore, the medical utilization characteristics were included as a continuous variable: the number of hospitalizations within one year, the total number of days of hospitalization of household members, admission to a long-term care hospital (yes or no), primary diagnosis for admission was neurologic disease (yes or no), cardiovascular disease (yes or no), hematologic or oncologic disease (yes or no), and musculoskeletal disease (yes or no).

### Statistical analysis

To confirm the association between the type of caregiving and catastrophic health expenditure, the covariates were compared using the chi-square test and t-test. Multiple logistic regression analysis was used to evaluate the association between the caregiving type and catastrophic health expenditure. The results were reported as odds ratios (ORs) and confidence intervals (CIs). Sensitivity analyses were conducted to evaluate the association between CHE and caregiving type by estimating the informal caregiving cost by calculating costs using opportunity and alternative cost approaches for ensuring comprehensive range of estimates. Model fitting was performed using the PROC SURVEYLOGISTIC procedure. All analyses were conducted using the SAS software, version 9.3 (SAS Institute, Cary, NC, USA).

## Results

Table 1 presents the characteristics of the study population, with a CHE threshold level of 40%. Of the 1,126 households, 70 (6.2%) households belonged to the “formal caregiver” group, of which 39 (55.7%) had a CHE threshold level of 40%. Furthermore, 70 (6.2%) households belonged to the “comprehensive nursing service” group, of which 18 (25.7%) had a CHE threshold level of 40%. A total of 986 (87.6%) households belonged to the “informal caregiver” group, 157 (15.9%) of which had a CHE threshold level of 40%.

**Table 1:** General characteristics of the study population (2019)

Variables	Catastrophic health expenditure						
	**Total**		**Yes**		**No**		
	**N**	**%**^**a**^	**N**	**%**^**b**^	**N**	**%**^**b**^	***P*-value**
**Total**	**1,126**	**100.0**	**214**	**19.0**	**912**	**81.0**	
**Type of caregiver**							< .0001
Informal caregiver	986	87.6	157	15.9	829	84.1	
Comprehensive nursing service	70	6.2	18	25.7	52	74.3	
Formal caregiver	70	6.2	39	55.7	31	44.3	
**Household head's sex**							0.0051
Male	866	76.9	147	17.0	719	83.0	
Female	260	23.1	67	25.8	193	74.2	
**Household head's age**							< .0001
less than 65	449	39.9	21	4.7	428	95.3	
65–74	276	24.5	68	24.6	208	75.4	
more than 75	401	35.6	125	31.2	276	68.8	
**Household head's employment status**							< .0001
Paid worker	429	38.1	32	7.5	397	92.5	
Self-employed worker	282	25.0	58	20.6	224	79.4	
others^a^	415	36.9	124	29.9	291	70.1	
**Household's Income level**							< .0001
low	274	24.3	93	33.9	181	66.1	
low-middle	341	30.3	93	27.3	248	72.7	
middle-high	265	23.5	21	7.9	244	92.1	
high	246	21.8	7	2.8	239	97.2	
**Region**							0.0064
Urban	460	40.9	76	16.5	384	83.5	
Rural	666	59.1	138	20.7	528	79.3	
**Number of household members**							< .0001
1 person	151	13.4	58	38.4	93	61.6	
2 persons	513	45.6	133	25.9	380	74.1	
over 3 persons	462	41.0	23	5.0	439	95.0	
**Medical-aid benficiary**							0.9603
Yes	67	6.0	16	23.9	51	76.1	
No	1059	94.0	198	18.7	861	81.3	
**Having a member with elderly ≥ 65**							< .0001
Yes	729	64.7	201	27.6	528	72.4	
No	397	35.3	13	3.3	384	96.7	
**Admission to long term care hospital**							0.0113
Yes	19	1.7	12	63.2	7	36.8	
No	1107	98.3	202	18.2	905	81.8	
**Primary diagnosis for admission**							
**Neuologic**							0.0019
Yes	99	8.8	24	24.2	75	75.8	
No	1027	91.2	190	18.5	837	81.5	
**Cardiovascular**							0.0208
Yes	35	3.1	11	31.4	24	68.6	
No	1091	96.9	203	18.6	888	81.4	
**Hematologic & oncologic**							< .0001
Yes	99	8.8	35	35.4	64	64.6	
No	1027	91.2	179	17.4	848	82.6	
**Musculoskeletal**							< .0001
Yes	241	21.4	81	33.6	160	66.4	
No	885	78.6	133	15.0	752	85.0	
**The number of hospitalization (mean/SD)**	1127	100.0	2.3	2.1	1.6	2.8	< .0001
**The total days of hospitalization (mean/SD)**	1127	100.0	35.1	52.1	12.8	17.6	< .0001

^a^column percentage, b: row percentage

Table 2 shows the association between the caregiver type and CHE. Those who received formal caregiving had an increased likelihood of CHE at threshold levels of 40% compared to those who received care from family (formal caregiver: OR, 3.11; CI, 1.63–5.92). Compared to those who received formal caregivers, households that used CNS had a decreased likelihood of CHE (CNS: OR, 0.35; CI, 0.15–0.82).

**Table 2:** Association between type of caregiver and catastrophic health expenditure

Variables	Catastrophic health expenditure			
	**OR**	**95% CI**		
**Type of caregiver (group 1)**				
Informal caregiver	1.00			
Formal caregiver	3.11	(1.63	-	5.92)
**Type of caregiver (group 2)**				
Informal caregiver	1.00			
Comprehensive nursing service	1.08	(0.55	-	2.12)
**Type of caregiver (group 3)**				
Formal caregiver	1.00			
Comprehensive nursing service	0.35	(0.15	-	0.82)

Adjusted all covariates

**Fig. 1:**
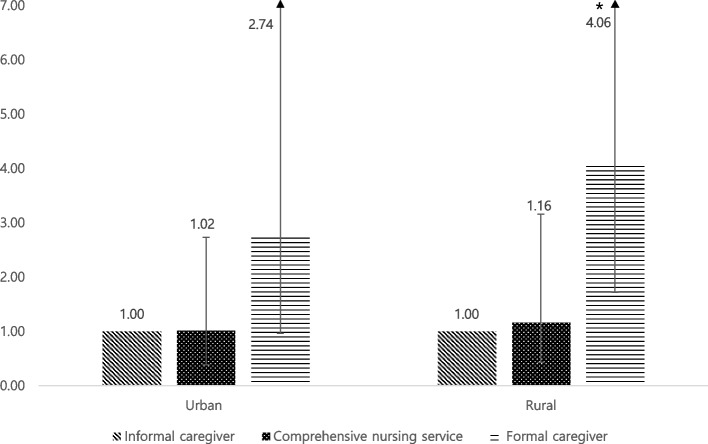
Association between type of caregiver and catastrophic health expenditure stratified by region. All covariates adjusted ^*^p-value<0.05

Figure 1 shows the association between caregiver type and CHE by region. Among households living in rural areas, those who received formal caregiving had an increased likelihood of CHE compared to those who received informal caregiving (formal caregiver: OR, 4.06; CI, 1.72–9.62).

Table 3 presents the results of the subgroup analysis to assess the association between CHE and caregiving type. Households that received only formal caregiving in a year were more strongly associated with CHE than those that only received care from family (only formal caregiver: OR, 3.30; CI, 1.57–6.92).

**Table 3:** Subgroup analysis stratified by type of caregiver

Variables	Catastrophic health expenditure			
	**OR**	**95% CI**		
**Type of caregiving**				
Only Informal caregiver	1.00			
Only Comprehensive nursing service	0.98	(0.46	-	2.09)
Only Formal caregiver	3.30	(1.57	-	6.92)
Use more than 2 types of services^a^	2.42	(0.94	-	6.27)

Adjusted all covariates

^a^Households using two or more of the three types of private care are included

Table 4 shows the sensitivity analysis of the association between CHE and the caregiving type by estimating the informal caregiving cost. In Model 1, the informal care cost was calculated by applying the average annual cost of formal caregivers using the replacement cost method. Households that received CNS had a decreased likelihood of CHE compared to those that received formal or informal caregiving. (group 2: OR, 0.43; CI, 0.21–0.89, group 3: OR, 0.37; CI, 0.14–0.97). In Model 2, the informal care cost was calculated using the opportunity cost method by applying the annual reservation wage rate. Compared to those who received formal or informal caregiving, households that received CNS had a decreased likelihood of CHE, however, this was not significant.

**Table 4:** Sensitivity analysis by estimated cost for informal caregiver

Variables	Catastrophic health expenditure			
	**OR**	**95% CI**		
**Sensitivity analysis model 1: informal caregiver cost, estimated by applying the market price of equivalent services**				
**Type of caregiver (group 1)**				
Informal caregiver	1.00			
Formal caregiver	1.16	(0.56	-	2.40)
**Type of caregiver (group 2)**				
Informal caregiver	1.00			
Comprehensive nursing service	0.43	(0.21	-	0.89)
**Type of caregiver (group 3)**				
Formal caregiver	1.00			
Comprehensive nursing service	0.37	(0.14	-	0.97)
**Sensitivity analysis model 2: informal caregiver cost, estimated reservation wage rate**				
**Type of caregiver (group 1)**				
Informal caregiver	1.00			
Formal caregiver	1.28	(0.63	-	2.60)
**Type of caregiver (group 2)**				
Informal caregiver	1.00			
Comprehensive nursing service	0.54	(0.26	-	1.10)
**Type of caregiver (group 3)**				
Formal caregiver	1.00			
Comprehensive nursing service	0.42	(0.16	-	1.08)

Adjusted all covariates

## Discussion

This study investigated the association between caregiver type and CHE at the household level among households utilizing inpatient medical services. Households receiving care from formal caregivers had a higher likelihood of CHE than households using informal caregivers. Compared to households using formal caregivers, those who used CNS had a lower association with CHE. The association with CHE differed based on the caregiving type used by each household.

Previous studies have shown that the burden of caregiving is increasing in many countries. The present study’s results showed that utilizing informal caregivers, rather than formal caregivers, reduced the risk of CHE. This can be explained in three ways: first, family caregivers of ill family members are mostly middle-aged or older women who may not have previously engaged in socioeconomic activities [24]**.** Therefore, it would not have affected previous household income. However, the financial burden of caregivers in an ageing society, which requires more caregiving, will be greater in the future due to the next generation of women being actively engaged in socioeconomic participation. Second, the cost of official caregivers in Korea is approximately $100 per day, [14] which is entirely an out-of-pocket cost, and there are not enough policies or insurance supporting this. Public long-term care insurance, financed by a new compulsory social insurance system, was introduced in South Korea in 2008 [25]. However, it supports the utilization of long-term facilities and home care among the eligibility of the service.

When examining policies related to alleviating the burden of caregivers in other countries, the policies did not focus on easing the burden of care for inpatients or the economic burden for paid caregivers. For example, the United States has two main federal policies providing workplace protection to caregivers with qualifications, the Americans with Disabilities Act (ADA) and the Family Medical Leave Act (FMLA). According to the ADA, employers cannot discriminate against caregivers caring for ill family members. Under the FMLA, employees with caregiving responsibilities for a family member are protected by taking unpaid leave for a period of 12 weeks and returning to their job, if they meet the specific criteria for hours worked and tenure [26]. The reason other countries focus on family care is that they do not need caregivers for acute hospital admissions. Other countries, such as the United States and European countries, established nursing and care services for inpatients in acute medical facilities as the role of nurses and established various systems to provide high-quality nursing and care services, such as service quality evaluation [27].

CNS was introduced in Korea in 2015 to realize a nurse-centered care system. This study showed that households that received CNS had a lower risk of CHE than those that used formal or informal caregiving services. This might provide evidence that this system can alleviate the economic burden of private care for inpatients. However, there is a possibility that the proportion of mildly ill patients among inpatients is high, consequently, CNS was initially expanded to include hospitals [28–30]. Eight years have passed since the policy was implemented, however, only 42.1% of all hospitals in Korea and 27.5% of all beds provided CNS. This study’s results also show that only 6.2% of households have experienced CNS [31]. According to previous studies, it has locally expanded disproportionately owing to the supply and demand problem of nursing personnel [32]. In this study’s results, individuals living in rural areas had a high risk when making use of formal caregiving. This might be due to the income of individuals living in rural areas being lower than that of people living in urban areas, and the higher proportion of older adults in rural areas. Therefore, CNS is considered particularly necessary in rural areas.

This study’s results indicated a strong association between households using informal caregivers and CHE among households receiving long-term care, compared to households using formal caregivers. The total days of care were strongly associated with the disease’s severity or the treatment’s intensity. Previous studies found that caregivers may experience a financial burden disproportionately relative to other caregivers due to the intensity of care they provide and the cost and complexity of treatment [26, 33, 34]. For example, the costs of caregiving increased significantly with increasing seizure frequency among patients with epilepsy [10]. Additionally, each stage of Alzheimer’s disease results in different responsibilities for caregivers, increasing their burden [33].

This study has some limitations. First, memory decay may be present, which may have interfered with each household’s complete healthcare expenditure and caregiving costs, as the KHP survey collected data through self-reports. While other data sources used for research on CHE in Korea have similar limitations, this study used the KHP dataset, which also used the complementary diary studies method to reduce recall bias and provided comprehensive information as a dataset specialized in health service use and expenditure. Second, this study was based on data from a cross-sectional study. Therefore, although associations could be confirmed, causality could not be evaluated. Third, owing to data restrictions, the opportunity costs of loss of productivity when using informal caregivers could not be estimated. Since this study’s data only covered one year, future data and research are required to evaluate the change in income. Fourth, the household level was analyzed, which did not fully capture the individual characteristics of illnesses, treatments, and length of hospital stay. However, this study considered the total sum of the length of hospital stay for family members and the sum of the number of hospitalizations of family members.

## Conclusions

This study investigated the association between caregiver type and CHE at the household level among households utilizing inpatient medical services. Households using CNS were likely to have a decreased association with CHE, compared to households using formal or informal caregiving. These findings highlight the need to support households forced to use formal caregivers.

## Supplementary Information

**Additional file 1: Appendix figure 1.** Flow chart.

**Additional file 2: Appendix table 1.** Association between type of caregiver and catastrophic health expenditure.

## Data Availability

The Korea Health Panel is secondary data. The KHPS is a national public database (https://www.khp.re.kr) that includes an identification number for each household and each member; however, the number is not associated with any personal identifying information, and the data collection system and database were designed to protect respondent confidentiality. Respondents were required to read and sign an agreement form before participating in the KHPS and to consent that their data could be used in future scientific research.

## Abbreviations

CHE: Catastrophic health expenditure
CNS: Comprehensive nursing services
KHP: Korean Health Panel
WHO: World Health Organization
OOPHE: Out-of-pocket health expenditure
ADA: Americans with Disabilities Act
FMLA: Family Medical Leave Act
