# Supplementary figures and images for: Association between caregiver type and catastrophic health expenditure among households using inpatient medical services: using Korean health panel
Source: BMC Health Serv Res. 2023 Jul 3;23:721. doi: 10.1186/s12913-023-09703-1 (PMC10316533; doi:10.1186/s12913-023-09703-1)

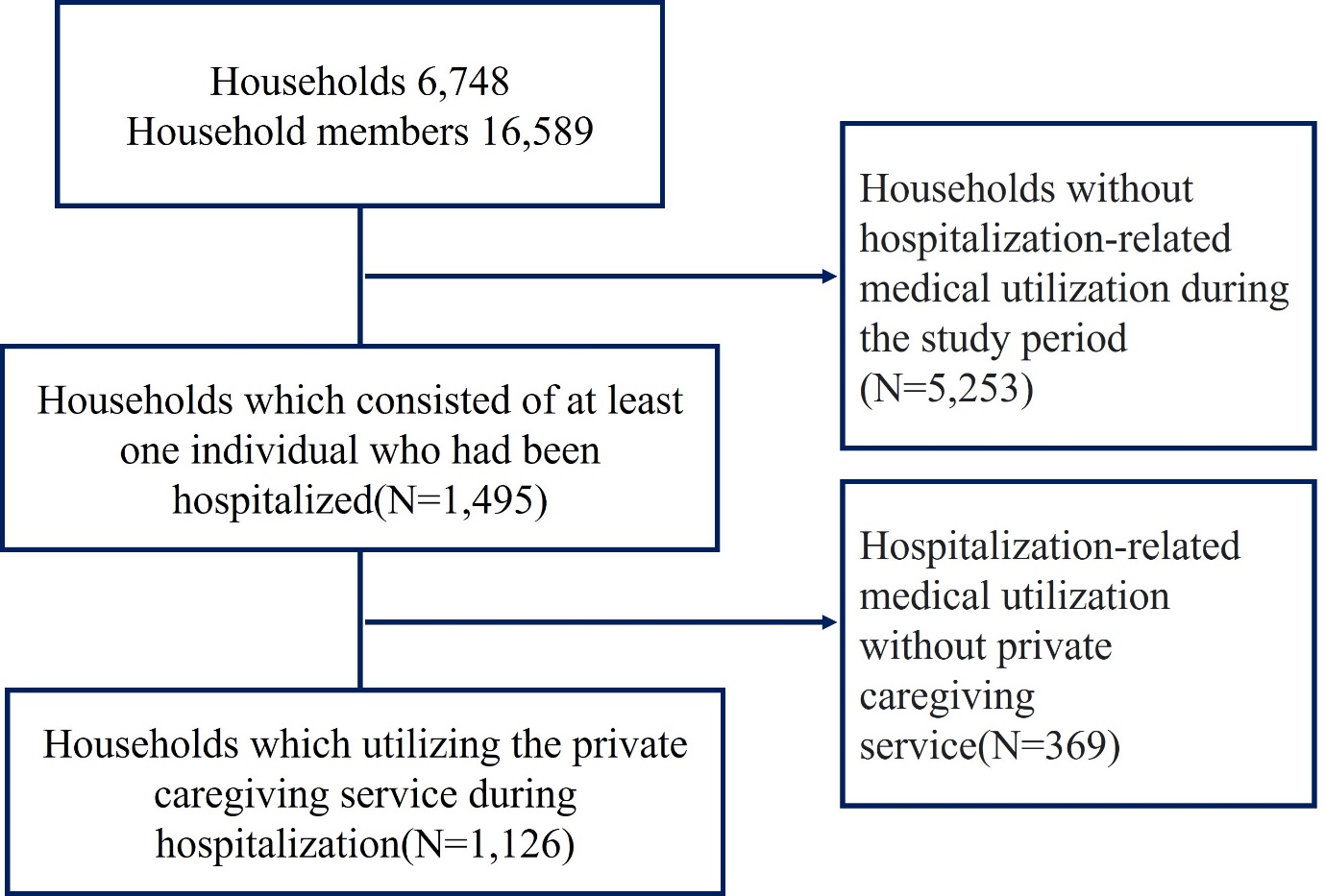


**Appendix figure 1. Flow chart**

Supplement: Supplementary file 1 — Additional file 1: Appendix figure 1. Flow chart. [file 12913_2023_9703_MOESM1_ESM.docx]
