# Supplementary material for: Association between caregiver type and catastrophic health expenditure among households using inpatient medical services: using Korean health panel
Source: BMC Health Serv Res. 2023 Jul 3;23:721. doi: 10.1186/s12913-023-09703-1 (PMC10316533; doi:10.1186/s12913-023-09703-1)
Supplement: Supplementary file 2 — Additional file 2: Appendix table 1. Association between type of caregiver and catastrophic health expenditure. [file 12913_2023_9703_MOESM2_ESM.docx]

| **Appendix table 1. Association between type of caregiver and catastrophic health expenditure** | | | | |
| --- | --- | --- | --- | --- |
| **Variables** | **Catastrophic health expenditure** | | | |
|  | **OR** | **95% CI** | | |
| **Household head's sex** | | |  |  |
| Male | 1.00 |  |  |  |
| Female | 0.52 | (0.30 | - | 0.90) |
| **Household head's age** | | |  |  |
| less than 65 | 1.00 |  |  |  |
| 65-74 | 0.69 | (0.22 | - | 2.15) |
| more than 75 | 0.71 | (0.23 | - | 2.19) |
| **Household head's employment status** | | | |  |
| Paid worker | 1.00 |  |  |  |
| Self-employed worker | 1.43 | (0.81 | - | 2.53) |
| others^a^ | 1.25 | (0.74 | - | 2.12) |
| **Household's Income level** | | |  |  |
| low | 15.75 | (5.27 | - | 47.06) |
| low-middle | 8.56 | (3.05 | - | 24.02) |
| middle-high | 2.43 | (0.83 | - | 7.18) |
| high | 1.00 |  |  |  |
| **Region** |  |  |  |  |
| Urban | 1.00 |  |  |  |
| Rural | 1.22 | (0.82 | - | 1.82) |
| **Number of household members** | | |  |  |
| 1 person | 1.00 |  |  |  |
| 2 persons | 0.62 | (0.34 | - | 1.15) |
| over 3 persons | 0.20 | (0.09 | - | 0.45) |
| **Medical-aid benficiary** | | |  |  |
| Yes | 1.00 |  |  |  |
| No | 2.28 | (1.06 | - | 4.88) |
| **Having a member with elderly ≥65** | | | |  |
| Yes | 2.64 | (0.79 | - | 8.86) |
| No | 1.00 |  |  |  |
| **Admission to long term care hospital** | | | |  |
| Yes | 2.59 | (0.62 | - | 10.80) |
| No | 1.00 |  |  |  |
| **Primary diagnosis for admission** | | |  |  |
| **Neuologic** |  |  |  |  |
| Yes | 1.32 | (0.54 | - | 3.22) |
| No | 1.00 |  |  |  |
| **Cardiovascular** |  |  |  |  |
| Yes | 1.31 | (0.72 | - | 2.38) |
| No | 1.00 |  |  |  |
| **Hematologic & oncologic** |  |  |  |  |
| Yes | 3.05 | (1.74 | - | 5.37) |
| No | 1.00 |  |  |  |
| **Musculoskeletal** |  |  |  |  |
| Yes | 3.23 | (2.11 | - | 4.95) |
| No | 1.00 |  |  |  |
| **The number of hospitalization** | 1.03 | (1.02 | - | 1.04) |
| **The total days of hospitalization** | 1.00 | (0.92 | - | 1.08) |
